# Supplementary material for: Splice-Junction-Based Mapping of Alternative Isoforms in the Human Proteome
Source: Cell Rep. Author manuscript; Available in PMC 2020 Jan 15. (PMC6961840; doi:10.1016/j.celrep.2019.11.026)
Supplement: 2 [file NIHMS1546469-supplement-2.zip › DF1/PXD009737/TestisLysC/TestisLysC_18_LRCH4_GTVEEQRPPELSPGAGDRERAPSSSGSPK.pdf]

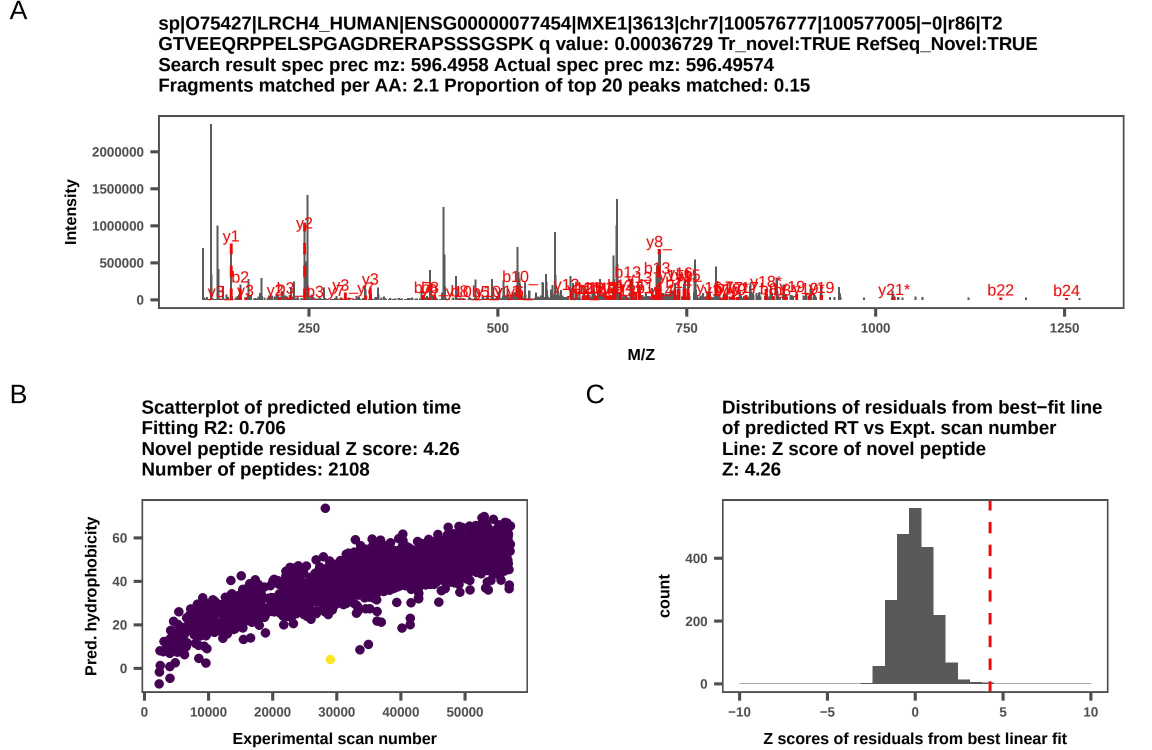
A
sp|O75427|LRCH4_HUMAN|ENSG00000077454|MXE1|3613|chr7|100576777|100577005|−0|r86|T2
GTVEEQRPPELSPGAGDRERAPSSSGSPK q value: 0.00036729 Tr_novel:TRUE RefSeq_Novel:TRUE
Search result spec prec mz: 596.4958 Actual spec prec mz: 596.49574
Fragments matched per AA: 2.1 Proportion of top 20 peaks matched: 0.15
Intensity
2000000
1500000
1000000
500000
0
y1
y2
b2
y8_
b10_
y21*
b22
b24
250
500
750
1000
1250
M/Z
B
Scatterplot of predicted elution time
Fitting R2: 0.706
Novel peptide residual Z score: 4.26
Number of peptides: 2108
Pred. hydrophobicity
60
40
20
0
0
10000
20000
30000
40000
50000
Experimental scan number
C
Distributions of residuals from best−fit line
of predicted RT vs Expt. scan number
Line: Z score of novel peptide
Z: 4.26
count
400
200
0
−10
−5
0
5
10
Z scores of residuals from best linear fit
